# Integration of Spirituality in Medical Education in Iran: A Qualitative Exploration of Requirements

**DOI:** 10.1155/2015/793085

**Published:** 2015-11-24

**Authors:** Nadereh Memaryan, Maryam Rassouli, Seyedeh Zahra Nahardani, Parisa Amiri

**Affiliations:** ^1^Academy of Medical Sciences of Islamic Republic of Iran, School of Behavioral Sciences and Mental Health, Iran University of Medical Sciences, Tehran, Iran; ^2^Pediatric Nursing Department, School of Nursing & Midwifery, Shahid Beheshti University of Medical Sciences, Vali-e Asr Street, Cross Niyayesh Highway, Tehran 1985717443, Iran; ^3^Department of Medical Education, Faculty of Medicine, Iran University of Medical Sciences, Tehran, Iran; ^4^Research Center for Social Determinants of Endocrine Health & Obesity Research Center, Research Institute for Endocrine Sciences, Shahid Beheshti University of Medical Sciences, Tehran, Iran

## Abstract

*Background*. Healthcare system has needed to provide spiritual services, and one of the reasons for not addressing spirituality in this field is lack of training in this area. This study purpose is to explore and identify main requirements for designing this education, in Iran.* Materials and Methods*. This is a qualitative study with conventional content analysis method. 18 participants, who were main stakeholders in spirituality, medical education, and curriculum development, were selected by purposive sampling. Data were collected using semistructured interviews, which continued until data saturation.* Results*. Three main themes and their categories were extracted from analysis of data. The themes are (1) educational needs including clinical practice needs; (2) opportunities including rich background and backup, perceived clinical need, and right context of medical education for change; and (3) challenges including challenges in academic planning and barriers to implementation.* Conclusion*. All stakeholders acknowledged the need for addressing spirituality in formal medical education. It seems that implementation of such programs requires attention to facilitating factors and challenges proposed by those involved.

## 1. Introduction

For many years, healthcare system has needed to provide spiritual answers and attend to this aspect of patients' health [1]. This is confirmed by studies that reveal patient needs in this area [2–5].

Spirituality has been addressed in Iranian traditional medicine in that great Persian physicians, like al-Razi and Avicenna, considered it an effective factor in health, and Iranian traditional medicine utilized this concept for years [6]. Also in recent years Iran is among countries active in the relevant science production and is sixth in terms of publications regarding health and spirituality [7]. However, there is no evidence showing that these studies have resulted in treatment and care programs in the health care system. In contrast, there are studies indicating the barriers to providing these services [8].

One of the reasons for not addressing spirituality in the field of health services is lack of training in this area [9]. Thus, providing such training appears essential [10].

Accordingly, inclusion and integration of spirituality in existing curricula has long been emphasized as a serious requirement [11] and has been seen in medical education documentations [12].

Despite the importance of this issue and the existence of relevant values in the strategic plans in Iran, it is still ignored in practice, so that no trace of spirituality-related education is currently observed in medical education in Iran [13].

In the US, the number of medical schools addressing such training increased from 3 in 1993 to 100 by 2011 [14]. The National Institute for Healthcare Research (NIHR) dedicated grants to 19 medical schools to develop a curriculum in spirituality and medicine in the USA in 1994. In the first year, 17 schools successfully received it and within two years 29 schools received it. Although medical schools were first different in theoretical frameworks and some components of their curricula, they had a few items in common: teaching spiritual assessment and spiritual history taking and attracting students' attention to this issue, the role of spirituality in health services based on research, religious counsellors and their role as the members of the treatment team, and emphasizing effective communication between therapists and patients even when patients are on the verge of death and the routine treatment is not effective [15].

However, development of such training is subject to finding a cohesive framework [16] that corresponds to regional and national requirements of each country [17].

Therefore, the general pattern of such education can be obtained through the review of the literature, but the key point of modifying the current curriculum of Iranian medical students which is suitable for this country is use of investigations with various methods, including qualitative studies [18].

Main stakeholders views are essential for educational policy making and successful implementation of these policies [19].

Since requirements for design and implementation of spiritual health curriculum have not yet been identified in Iran [20] the purpose of this study is to explore and identify main requirements for designing this program through stakeholders' views.

## 2. Materials and Methods

This qualitative study with conventional content analysis method was conducted to identify main requirements, through a deductive approach. This approach is employed when the researcher does not intend to use presumptions in analysis of data [21]. Study population comprised main stakeholders of the curriculum including target audience (students and teachers), eventual stakeholders (recipients of health services), religious experts, medical education experts (former members of the Supreme Council for Medical Education Planning), and executives of educational programs (departmental managers, university chancellors, and health services personnel), of whom participants were selected.

### 2.1. Data Collection

Participants were selected by purposive sampling, which continued until data saturation. Data were collected using semistructured interviews based on the developed framework. A total of 18 interviews were conducted with 4 students, 10 faculty members (with former roles as university chancellors, school deans, departmental managers, and members of the Supreme Council for Planning), 2 religious experts (with Ph.D.), and 2 health service providers (various activities of participating medical professionals are also considered as part of health service providers). Interviews lasted from 30 minutes to 95 minutes, were recorded with written permission of participants, transcribed verbatim, and turned into computer files.

### 2.2. Analysis of Data

Data were analyzed according to conventional content analysis approach [22]. Transcribed interviews were considered as analysis units. Meaning units were extracted from interview texts, after several times of reading and understanding sentences. Codes were formed that may be referred to as labels for meaning units. Next, categories were created from grouping of similar codes. Theme is the foundation for contents in categories [23, 24].

### 2.3. Data Trustworthiness

As Guba and Lincoln's Evaluative Criteria [25], trustworthiness and rigor of data were achieved through prolonged engagement and immersion, peer check, and data source triangulation (use of experts in various fields in collection of data) and external check (to confirm rigor of extracted codes).

### 2.4. Ethical Considerations

This study was approved by the Research Council of the Academy of Medical Sciences of Islamic Republic of Iran (27th session of research group dated 20.10.2013). Prior to every interview, written consent for participation was obtained after briefing about the study objectives, confidentiality of data, anonymous publication of results, and the right to withdraw at any stage. Ethical principles and respect for participants' rights were observed in the course of every interview.

## 3. Results

Of the 18 participants, 12(67%) were men and 6(33%) were women. Field of education and activity of participants are presented in Table 1. Three main themes emerged from analysis of data and categories created, including educational needs (Table 2), capacities (Table 3), and challenges (Table 4); each is addressed as follows.

### 3.1. Educational Needs

This was the first theme extracted from data and involves educational needs with respect to inclusion of spirituality in medical sciences curricula and contains three categories of needs: cognitive, emotional, and psychomotor needs.

#### 3.1.1. Cognitive Needs

In cognitive area, educational needs are rather connected with concepts, principles, indicators, and components of spiritual health. One of the participants remarked:* “The first thing that comes to mind is what the term spirituality means, and what difference it has from other terms?”* (P2, medical student). This category also includes pathology and knowledge of interventions recommended to eliminate damages, including:* “You should realize that in essence, principles of medical science are based on the subject of pathology. Isn't spiritual health part of health?”* (P15, Ph.D. in religious sciences). According to participants, with regard to cognitive needs, the need to specifically define spiritual interventions is also felt:* “We should recommend the subject to students, and after every topic, one chapter should be allocated to this subject”* (P16, faculty member).

#### 3.1.2. Emotional Needs

Codes associated with this category encompass importance of spiritual dimension of mankind:* “A medical student should first arrive at the realization that spiritual health forms an extremely important dimension of health, and probably includes physical, psychological, and social health”* (P18, clinical subspecialist with teaching and research background at national and international levels). According to participants, it is hugely important for stakeholders to realize that the issue has been subject of research throughout the world for a long time. For instance, a medical subspecialist and faculty member argued:* “I have a copy of guidelines from other countries, and I can show it. Suppose you log onto Australian Islamic Council site; you will see all our guidelines there, including our tayammum soil, which you cannot find in our own sites.”*

#### 3.1.3. Psychomotor Needs

From the perspective of stakeholders, the most important skill medical groups need to acquire is in the area of diagnosis:* “Naturally, I'm not supposed to treat everything. It's the same in every branch of science. But we are expected to diagnose. For instance, when a patient comes in, a GP ought to be able to diagnose his problem”* (P12, Ph.D. in religious sciences). One of the most important diagnostic trainings is taking spiritual history and separation of disorders in different dimensions:* “Then, teaching interviewing skills, how to diagnose their pathologies, and differentiate between different areas”* (P13, Clinician, with many experiences in teaching and department and school management).

Another participant, a leading spiritual service provider in health centers across the country stated:* “I believe first, one, two or three basic spiritual questions about assessing spiritual dimensions of the patients should be included in curriculum of all medical groups”* (P17). Some experts were of the opinion that elementary and simple interventions and care can be taught and employed:* “If you were told that you did not need to do much, only hold her hand and tell her: ‘Mother! I understand'. That's not much, and swear to God it is not hard, but helps. Yet, we close our eyes and do not want to see”* (P9, Nursing Ph.D., faculty member, health service provider). Also, when intervention is beyond her capability and she needs to refer the case to a specialist:* “She should be able to realize, if not her, then whom and where? And refer patient to where he should go”* (P12, religious science Ph.D.).

### 3.2. Capacities

This theme addresses existing strengths, capacities, and opportunities in the issue of inclusion of spirituality in national medical sciences curriculum and includes categories of rich background and support, perceived clinical need, and the right medical education context for change.

#### 3.2.1. Rich Background and Backup

Participants' statements suggest existence of rich sources in Iran for spirituality. A participant (medical education postgraduate student) asserted:* “We have always had many rich things in our religious documents for reaching spiritual and religious health”* (P6).

#### 3.2.2. Perceived Clinical Need

The emphasis here is on patients' spiritual needs and their demand for receiving such care. Attention is also paid to several clinical guidelines for responding to these needs.* “In my work, I've been very much in touch with spirituality. I've worked in ICU for 10 years, where I saw spirituality with my own eyes”* (P5, senior nursing expert, health service provider). Final year medical student (P10) argues:* “So many times treatment is not effective and you reach a dead end, and patient is not satisfied, and most patients need such a thing.”*

#### 3.2.3. Right Medical Education Context for Change

Citing predisposing context in medical education for inclusion of spirituality in existing curricula, participants talked about some of the evidence. Subjects already predisposed to inclusion of spirituality (including knowledge and ethics courses) are considered as a pretext in medical education. Moreover, legal supports and constant changes in medical sciences courses have accustomed this field to change. One of the participants with several years' experience in educational posts across the country and further studies in this area (P8) stated: “*Previously, one reference book was used for many years, without change. But now, same books are revised in a short while, and some topics are changed, and others may become relevant according to needs and socioeconomic and political developments. Thus, generally, change and development in medical sciences is a permanent necessity, and should be carried out continuously. The same is seen in development programs. All programs are planned in accordance with the Supreme Council and the Ministries of Health and Education with a legal requirement to be carried out.”*

### 3.3. Challenges

Another theme extracted was challenges threatening inclusion of spirituality in medical education curricula and includes categories of challenges in academic planning and obstacles to implementation.

#### 3.3.1. Challenges in Academic Planning

This category addresses challenges in academic planning. For instance, one of the challenges is lack of necessary content for inclusion in medical education, which relates to the abstract nature of spirituality and experimental and material nature of medicine, making it difficult to combine the two. Meanwhile, no consensus has yet been reached on the definition of spiritual health. Thus, no standard intervention and care model has yet been devised in accordance with the context of Iranian society. A faculty member stated:* “What intervention do you want to do for a vague thing like spiritual health? We have no specific model for our Muslim people with their own particular cultural beliefs”* (P1).

#### 3.3.2. Barriers to Implementation

According to participants' statements, it seems that most challenges are associated with implementation of curriculum and necessary contexts, including saturation of medical education curricula and lack of room for inclusion of a new subject. An educational official of the Ministry of Health said:* “Right now, we have 60 units, each with its own place. If we were to include all this, we would be short of units. We have a limit”* (P14). Another issue raised was the need for manpower to train specialists in spiritual therapy and acceptance of referrals.

## 4. Discussion

This study explored the main requirements for designing spiritual curricula in medical education through stakeholders' views. Results obtained identified educational needs, capacities, and challenges for integration of spirituality in medical training course.

The theme of educational needs emerged from combination of three categories of clinical practice needs, which agrees with areas associated with educational goals devised by Bloom [26]. Identifying and presenting educational needs is considered an introduction to designing curriculum and determining capabilities required by medical students. Effort should be made to empower students in all clinical practice areas [27].

Extracted educational needs, with subsequent educational expectations from students, are in line with competencies published under National Competencies in Spirituality and Health for Medical Education [16], which include definition of basic concepts, including spirituality and religion, reasons and importance of attention to spirituality in patient care, spiritual assessment, issues relating to spiritual care of patients, and referrals to religious experts.

Among other studies on educational needs in spirituality curriculum for patient care is a study designed at the University of Missouri-Kansas city [28]. This study also refers to spiritual philosophy and debate and exchange of views about this concept and emphasizes the importance and effects of spirituality on patient health and highlights the role of the clergy in providing spiritual services, which fully concurs with codes and concepts extracted in the present study in all three areas of educational needs.

In the present study, one of the most important skills that should be acquired by students was taking spiritual history and diagnosis, which is in line with activities of some medical schools throughout the world that have included this training in their curricula in various ways and have taught their students how to take spiritual history and the right way to deal with religious issues [14, 29]. One of the key objectives at the Association of American Medical Colleges (AAMC) is for students to learn how to take spiritual history as part of taking patient history and use that in diagnosis [29].

This study also assessed positive and negative capacities and opportunities for implementation of curriculum designed accordingly. Capacities partly referred to cultural and belief backgrounds in Iran, partly to services and clinical nature of providing health services, and partly to educational system and university capacities, which, as a whole, drew the needy and ready atmosphere for implementation. Challenges cited partly related to stakeholders' concerns about educational content, which is a main requirement for a successful curriculum, and partly related to operational problems and context needed for implementation. It should be noted that culture and belief in any community can act as a facilitator or inhibitor in integration of spiritual program in university curricula, and, as discussed, it is seen as a facilitator in Iran. In a study in Australia, one of the most important challenges to such plans was less importance of religious issues in Australian community [30].

A study conducted in Brazil proposed lack of time and awareness, lack of necessary training, and fear raising issues associated with patient because of his possible dislike as the most important barriers to implementation of such programs, and most of these barriers are attributed to medical schools' failure to provide appropriate training [11], which somewhat agrees with the present study findings. In other words, it can be concluded from studies in this area that noninclusion of spiritual issues in academic curricula of medical education is the most important challenge and barrier to providing such care, and, thus, the right strategy to overcome this issue is adjustment of curricula and inclusion of relevant content in them.

In the present study, although all stakeholders acknowledged the need for addressing spirituality in formal medical education, it seems that implementation of such programs requires attention to facilitating factors and challenges proposed by those involved; otherwise taking the first step in design and implementation will not be possible. Accordingly, it is recommended that a study explain strategies to overcome challenges in design and implementation of integration of spirituality in medical education.

Based on our knowledge, the study is the first study in Iran exploring challenges of including spirituality in medical education curriculum.

One of the limitations of this study was selecting participants from Tehran, the capital of Iran, although the most experts in this fields work in Iranian ministry of health as the main policy making center for medical education.

Since this study aimed to assess requirements of integration of spirituality in medical education, no interview was conducted with patients. However, it will be necessary to gather views of this group of stakeholders to assess operational requirement in following studies.

## 5. Conclusion

This qualitative study identified the educational needs of the integrating spirituality in the medical curricula and the necessity for reflecting the educational needs identified in the curriculum. Furthermore, it examined the capacities and challenges for designing such a curriculum and revealed that developing a proper curriculum in this area and implementing it properly require addressing the stakeholders of this curriculum, taking advantage of identified opportunities, and dealing with the challenges.

## Figures and Tables

**Table 1:** Participants' demographic details.

	Gender	Education field	Activity/position
1	Male	Clinician	Faculty
2	Male	General practitioner	Final course of studentship
3	Male	Ph.D., basic sciences	Educational-research
4	Female	Clinical subspecialty	Faculty
5	Female	M.S., nursing	Health service provider
6	Female	Medical education	Ph.D. student
7	Male	Ph.D., basic sciences	Research, university chancellor
8	Male	Clinician/medical education subspecialty	Membership in the Supreme Planning Council-education
9	Female	Ph.D., nursing	Faculty, health service provider
10	Female	General practitioner	Intern
11	Female	General practitioner	5th year student
12	Male	Ph.D., religious studies	Education-research
13	Male	Clinician	Department head, school dean
14	Male	Public health specialist	In charge of Medical Education Development Council, Ministry of Health
15	Male	Ph.D., religious studies	Education-research
16	Male	Medical ethics specialist	Education-research
17	Male	Basic sciences	Researcher in spiritual health, health service provider
18	Male	Clinical subspecialty	Education-research

**Table 2:** Codes and categories related to educational requirements theme.

Theme	Category	Codes
Educational requirements	Cognitive needs	Concept of spirituality and spiritual health
		Spiritual health in Islamic sources
		Indicators and criteria of spiritual health
		Concepts of religion
		Spiritual health assessment tools
		Spiritual pathology
		Spiritual treatments (interventions)
		Differentiating problems of different areas of health
	Emotional needs	Attention to, importance of, and priority of spiritual dimension of providing services
		Statement of problem and academic and up-to-date evidence
		Reasons and routes for entry of spirituality in clinical setting
	Psychomotor needs	Spiritual assessment (taking history)
		Principles of spiritual care of patients and care providers
		Intervention range (simple and advanced)
		Referral

**Table 3:** Codes and categories associated with capacities theme.

Theme	Category	Codes
Capacities	Rich background and backup	Profound need for spirituality and several experiences suggesting it
		National religious capacity
		Rich sources and prominent scholars
		Rich Islamic literature, especially shiite, in this area
	Perceived clinical need	Spiritual peace for patients
		Spiritual nature of treatment
		Intense need for spirituality in treatment
		Several experiences and guidance
		Better acceptance and tolerance of illness by spiritual people
		Simple and effective spiritual primary care
	Right context of medical education for change	Capacity for topics such as ethics and knowledge
		Medical science familiar with change and development
		Legal support for changes in educational system
		Evidence from reputable international universities

**Table 4:** Codes and categories associated with theme of challenges.

Theme	Category	Codes
Challenges	Challenges in academic planning	Lack of consensus on definition of spiritual health
		Intangible and abstract concept of spirituality
		Materialistic and experimental views in medicine
		Lack of domestic interventions and models
	Barriers to implementation	Lack of feeling of the need in many professors
		Spiritual care considered as interference uncalled for
		Clinicians not spending time
		Implementation by people other than program writers
		Overloaded curriculum
		Need for role-models and teachers
		Need for spiritual-therapy specialist
		Unpredictability of outcomes of implementation across the country
